# Alteration of cortical functional networks in mood disorders with resting-state electroencephalography

**DOI:** 10.1192/j.eurpsy.2024.532

**Published:** 2024-08-27

**Authors:** S. Shim

**Affiliations:** psychiatry, Soonchunhyang university hospital, cheonan, Korea, Republic Of

## Abstract

**Introduction:**

This study investigated source-level cortical functional networks using resting-state electroencephalography (EEG) in patients with Bipolar disorder and Major depressive disorder, comparing the neuropathology of these disorders.

**Objectives:**

This study investigated source-level cortical functional networks using resting-state electroencephalography (EEG) in patients with Bipolar disorder and Major depressive disorder, comparing the neuropathology of these disorders.

**Methods:**

A total of 116 participants (35 patients diagnosed with bipolar disorder(BD), 39 patients diagnosed with Major depressive disorder(MDD), and 42 people who are healthy-control groups(HC)) were enrolled for this study. Depression and anxiety were evaluated with using State-Trait Anxiety Inventory (STAI) and Beck Depression Inventory (BDI). Graph theory‑based source‑level weighted functional networks were assessed via strength, clustering coefficient (CC), and path length (PL) in six frequency bands.

**Results:**

At the global level, patients with BD and MDD showed higher strength (p = 0.001) and CC (p = 0.001), and lower PL (p < 0.001) in the high beta band, compared to HCs. At the nodal level, compared to HCs, patients with BD showed higher high beta band nodal CCs in the right precuneus(p < 0.001), left isthmus cingulate(p < 0.001), bilateral paracentral(p < 0.001), and left superior frontal(p < 0.001); however, patients with MDD showed higher nodal CC only in the right precuneus(p < 0.001) compared to HCs. Although both MDD and BD patients had similar global level network changes, they had different nodal level network changes compared to HCs.

**Conclusions:**

This study suggest that both patients have similar network changes at the global level, but they have different network changes at the nodal level. Also, the higher nodal CCs in the high beta band might indicate the regions became more connected with their neighbors in accordance with the severity of depressive and anxious states. This study found a significant correlation between cortical network state and anxiety-related psychological measure in BD patients. Our source-level cortical network indices might contribute to the understanding of the neuropathological mechanisms in these two disorders.

**Disclosure of Interest:**

None Declared

